# Emerging evidence of occipital nerve compression in unremitting head and neck pain

**DOI:** 10.1186/s10194-019-1023-y

**Published:** 2019-07-02

**Authors:** Pamela Blake, Rami Burstein

**Affiliations:** 1 University of Texas Health Science Center at Houston, 2711 Ferndale Street, Houston, TX 77098 USA; 2 Beth Israel Deaconess Medical Center, Harvard Medical School, Boston, MA USA

**Keywords:** Occipital nerve, Extracranial, Neck pain, Chronic migraine, Cervicogenic headache, Nerve compression, Nerve decompression surgery

## Abstract

Unremitting head and neck pain (UHNP) is a commonly encountered phenomenon in Headache Medicine and may be seen in the setting of many well-defined headache types. The prevalence of UHNP is not clear, and establishing the presence of UHNP may require careful questioning at repeated patient visits. The cause of UHNP in some patients may be compression of the lesser and greater occipital nerves by the posterior cervical muscles and their fascial attachments at the occipital ridge with subsequent local perineural inflammation. The resulting pain is typically in the sub-occipital and occipital location, and, via anatomic connections between extracranial and intracranial nerves, may radiate frontally to trigeminal-innervated areas of the head. Migraine-like features of photophobia and nausea may occur with frontal radiation. Occipital allodynia is common, as is spasm of the cervical muscles. Patients with UHNP may comprise a subgroup of Chronic Migraine, as well as of Chronic Tension-Type Headache, New Daily Persistent Headache and Cervicogenic Headache. Centrally acting membrane-stabilizing agents, which are often ineffective for CM, are similarly generally ineffective for UHNP. Extracranially-directed treatments such as occipital nerve blocks, cervical trigger point injections, botulinum toxin and monoclonal antibodies directed at calcitonin gene related peptide, which act primarily in the periphery, may provide more substantial relief for UHNP; additionally, decompression of the occipital nerves from muscular and fascial compression is effective for some patients, and may result in enduring pain relief. Further study is needed to determine the prevalence of UHNP, and to understand the role of occipital nerve compression in UHNP and of occipital nerve decompression surgery in chronic head and neck pain.

## Introduction

### Challenges in identification of unremitting head and neck pain

Unremitting head and/or neck pain (UHNP) is a commonly encountered phenomenon in clinical practice, although recognition of the symptom may prove challenging. Most questioning of patients that occurs during clinical visits is aimed at establishing the frequency of head pain, and while neck pain is a widely acknowledged common co-morbid feature of headache, the frequency and duration of pain in the neck is less often as carefully assessed as head pain. In the authors’ experience, UHNP often presents with constant mild discomfort, frequently described as ‘tightness’ or ‘pressure’ in the posterior neck and sub-occipital space, with less frequent, yet more intense bouts of pain radiating to the vertex, temples and forehead. Obtaining the history of unremitting pain may thus require careful questioning, as it is common for patients to be less aware of mild, yet constant discomfort in the neck, and to have greater awareness of the days of severe headache pain, particularly if that head pain is associated with migrainous features such as nausea and photophobia. It is also common, with very longstanding symptoms, for patients to even lack awareness of the presence of unremitting neck pain; such lack of awareness may be compounded in the presence of presence of conditions that interfere with the observation of or reporting of pain, such as attentional issues or cognitive inefficiencies. (Also referred to as cogniform condition, cognitive inefficiencies are cognitive difficulties that are due not to neurological conditions but rather to factors such as affective conditions, fatigue, or pain, and which can be elucidated with formal neuropsychological testing) [1]. The presence of unremitting pain in the head and/or neck may thus be missed, even by an experienced clinician, and directing the patient to the development of an awareness of how the head and neck feels on a daily basis, with additional history at follow-up visits, is often necessary to determine if UHNP is present. The effort is rewarded, however, as confirmation of the presence of UHNP may offer opportunities for effective intervention.

There is no epidemiologic data regarding the prevalence of constant headache or UHNP, as population studies of headache, in assessing headache frequency, typically employ the benchmark of 15 or more days per month required for the diagnoses of chronic migraine (CM) or chronic tension-type headache (CTTH), and do not distinguish unremitting pain as a distinct entity. There are, however, some suggestions in the epidemiologic data that a subset of patients with CM may in fact have UHNP. About 34% of patients with CM have persistent, very high frequency of headache (25–31 days per month), and such individuals are least likely to experience remission of CM to episodic migraine at subsequent follow-up [2]. Thus, a subset of patients with chronic migraine have very frequent headache that tends to persist. Other than this possible glimpse into epidemiologic data, the prevalence of UHNP is not known.

### Possible headache conditions associated with UHNP

The International Classification of Headache Disorders (ICHD) [3] offers several conditions that may be associated with UHNP, including CM, CTTH, New Daily Persistent Headache (NDPH), and Hemicrania Continua (HC), however, many patients with unremitting head and neck pain do not meet the criteria for any of these conditions. Patients with UHNP, for example, may not present with the abrupt onset of pain required for NDPH, nor with the strictly unilateral, indomethacin-responsive pain required for HC. Likewise, many patients with UHNP do not have pain that is the bilateral, mild-moderate intensity pain required for the diagnosis of CTTH, and many have pain that does not fully meet CM criteria, for instance, lacking the required minimum of 8 days per month of nausea and photophobia.

Challenges continue beyond the establishment of the presence of UHNP to the realms of diagnosis and treatment. The management of UHNP is unclear, in part because UHNP is generally not evaluated in clinical studies. There was no query regarding the presence of neck pain as a factor directly related to headache characteristic or frequency, or to headache reduction, in any of the clinical studies of the three commercially available monoclonal antibodies directed at calcitonin gene related peptide (CGRP), including erenumab [*personal communication, Jason Skinner, Phd, Amgen*], fremanezumab [*personal communication, Michael Seminerio, PhD, Teva Pharmaceutical*, or galcanezumab [*Iman Mangum, Lilly USA*], or in the clinical trials for botulinum toxin [*Harsha Patel, PhD, Allergan*]*.* Additionally, subjects with constant headache are typically excluded from clinical studies of botulinum toxin and of CGRP monoclonal antibodies, as such patients lack discrete episodes of headache that can be counted for statistical analysis [*personal communication, Michael Seminerio, PhD, Teva Pharmaceutical; Jason Skinner, Phd, Amgen; Iman Mangum, Lilly USA; Harsha Patel, PhD, Allergan*].

Critical in UHNP is the involvement of the neck. Neck pain is common in CTTH and migraine [4, 5], and the presence of chronic neck pain may in fact convey a particular clinical significance. The combined symptoms of CTTH, neck pain and migraine are not only common, representing 67% of patients with migraine evaluated in a tertiary headache clinic [6], but also convey a higher level of severity: these patients are the most afflicted, with the lowest rates of physical activity and psychological well-being and with higher levels of perceived stress [6]. Additionally, the presence of neck pain has been shown to correlate with increasing frequency of headaches [5].

The involvement of the neck may suggest the diagnosis of cervicogenic headache (CH) [3] in patients with UHNP. The diagnostic criteria for CH require ‘clinical and/or imaging evidence of a disorder or lesion within the cervical spine, known to be able to cause headache.’ While radiographically evident lesions involving the spine itself or the bony elements of the spinal column as a cause of headache tend to be uncommon in clinical practice, it is indeed possible for soft tissue lesions of the neck to be present and to cause pain, as will be discussed below. Other diagnostic criteria for CH address the temporal relationship between the ‘onset of the lesion’ and the onset of headache, and the parallel reduction in headache and any accompanying neck pain with elimination of the cervical lesion. Such experiences are also uncommon in the clinical setting, particularly as it is difficult to assign a time of onset to a lesion that cannot be diagnosed on imaging. It is notable, however, that the diagnostic criterion of reduced cervical range of motion and worsening of pain with provocative maneuvers is commonly encountered in patients with UHNP, as discussed below.

## Proposed mechanism for the origin of unremitting head and neck pain

### Lack of evidence of a central etiology of pain in headache

The etiology of pain in chronic headache, including UHNP, CM and CTTH, is not yet clear. While nociceptive activity of the meninges and the trigeminovascular system is well established, the underlying activators of these nociceptors is unclear. The etiology of neck pain is similarly unknown. Some authors have attributed the common occurrence of neck pain to the convergence of sensory afferents from the upper cervical region with trigeminal afferents in the trigeminal cervical complex [7]; this theory, however, remains unproven. The absence of imaging abnormalities of cervical bony and neural structures that are within the spatial resolution of current clinical MRI has further supported the concept that central processes involving upper cervical afferents along with trigeminal nociceptors is the cause of neck pain in headache. Thus, most of the research on the cause of headache pain has been directed at intracranial processes, such as central sensitization and activation of brainstem pathways [8], and the role of these central processes in migraine has accordingly become generally accepted and regarded as validated, even when the conclusions of research were not necessarily consistent with a causative, solely central process. For example, some findings in migraine that have been interpreted as suggestive of central causation, such as altered functional activity in the anterior cingulate cortex, prefrontal cortex, thalamus and somatosensory cortex [9], have also been reported in other chronic pain conditions, such as fibromyalgia [10]. Such findings may thus reflect alterations of pain processing that are common to many chronic pain conditions rather than indicating phenomena unique to migraine.

### The anatomy and pathophysiology of extracranial sources in pain

Given the absence of clear evidence of a central process causing UNHP, it is notable that several important findings within the past decade have raised the possibility that extracranial mechanisms may play an important role in headache generation. In 2009, it was reported that that nerve branches with both sensory (peripherin-positive) and pain (CGRP- and TRPV1-positive) functions emanated from the dura of mouse pups, traversed the calvarium and resided in the periosteum [11]. In adult mice with fully calcified calvaria, these meningeal fibers remained intact within the suture lines and the periosteum [11]. This study provided the first evidence of a direct anatomic connection between the intracranial and extracranial spaces and a possible role for an extracranial source of pain in headache. Four years later, the 2013 publication of anatomic studies of rat skulls demonstrated that the source of these calvarium-crossing nerves were the maxillary and mandibular branches of the trigeminal nerve [12]. These studies also shed light on the functional implication of this anatomy, demonstrating that stimulation of pericranial muscles resulted in the intracranial release of CGRP. The authors postulated that this connection between the extracranial and intracranial compartments may explain how noxious stimulation of pericranial tissues may influence meningeal nociception and thus headaches. In 2014, the same authors, using rat and human skulls, demonstrated via the application of tracer to trigeminal nerves that branches of these nerves also exit the skull through emissary canals and innervate not only the periosteum but also the deep layer of temporal and upper neck muscles. [13] Finally, it was recently been reported that nerve fibers arising from C2–3 dorsal root ganglia traverse the posterior cervical muscles and, passing through the cranium, innervate the dura of the posterior fossa [14]. The authors speculated that the origin of occipital headaches thus likely differs from that of frontal headaches, and that occipital headaches are more likely to be associated with cerebellar abnormalities. These studies together demonstrate extensive interconnections between the intracranial and extracranial spaces and establish anatomic and functional pathways by which an extracranial trigger in the head or neck can result in activation of intracranial meningeal nociceptors, giving rise not only to head pain but also to activation of central processes that may cause migraine-like symptoms including photophobia and nausea.

Absent from the above studies was evidence of an extracranial pathology that could stimulate the described nociceptive pathways. A 2016 study provided such evidence. Genetic studies of the occipital periosteum in 18 individuals with chronic headaches with prominent occipital pain demonstrated a significant increase in the expression of pro-inflammatory genes and decreased expression of genes that suppress inflammation in the occipital periosteum [15]. The authors theorized that inflammation of the molecular environment of pain fibers in the occipital periosteum could activate the trigeminal nociceptive fibers that reach the periosteum, a pathophysiology which may now be extended to upper cervical derived nerve root fibers that travel intracranially.

The presence of extracranial fibers arising from meningeal nociceptors, and the demonstration of extracranial pathology in the form of inflammation, may address the question as to why centrally-directed treatment for migraine are so often ineffective. The standard pharmacological treatment for CM or CTTH has traditionally been centrally acting agents that reduce neuronal excitability, such as anti-convulsants and anti-depressants [8]; however, adherence to such treatment regimens is low, indicating low efficacy, at least for some patients [16, 17]. Similarly, behavioral interventions such as Cognitive Behavioral Therapy, while highly effective for some individuals [18, 19], often do not provide adequate and prolonged headache reduction. As a result of inadequate efficacy of these centrally-directed treatments, several treatments directed at the extracranial portion of the head and neck have been increasingly incorporated into the practice of headache medicine over the last decade. Such treatments include occipital nerve blocks [20], trigger point injections [20], botulinum toxin injections [21], and more recently, the use of monoclonal antibodies targeting the calcitonin gene related peptide pathway acting mostly outside of the blood-brain barrier [22].

### The role of occipital nerves in headache

An additional, less widely employed intervention directed at extracranial potential pain generators is surgical decompression of the greater occipital nerve (GON) in its course through the posterior cervical muscles. This procedure was initially reported in 1992 [23] in a procedure conducted in 50 patients under local anesthesia, employing decompression of the GON from the overlying trapezius muscle; in many patients, compression was also noted at the semispinalis capitis muscle. Eighty-two percent of patients had some or complete relief immediately. At 14.5 months follow-up, 28 patients (56%) expressed benefit from the surgery with less or no post-operative disability. No one was worse after the surgery, and there was no operative morbidity. The authors indicated that failure to decompress at the semispinalis capitis muscle may have been the cause of the cases of recurrence of pain. A 1996 study [24], similarly conducted under local anesthesia, reported similar outcomes in 18 patients. In 2004, outcomes from a smaller study in ten patients, employing general anesthesia and a more comprehensive decompression of the nerves to the layer of the inferior oblique muscle, was published [25]. At a mean follow-up of 37 months the mean Visual Analog Scale pain score had decreased from the pre-operative mean score of 70 to a mean score or 20. Seven of the 10 patients were satisfied or very satisfied with the results of the procedure, and there were no intraoperative complications. These early studies were limited due to the lack of control groups and also to lack of classification of headache type, as no specific ICHD diagnoses were applied; nevertheless, the response to treatment and the lack of adverse effects was promising.

The mechanism by which ON compression may cause headache may lie in the resulting inflammation that has been observed in both animal and human studies of nerve compression. Experiments in the rat model [26, 27] have demonstrated inflammation both local to and remote from the site of peripheral nerve compression. Similar findings of local inflammation due to nerve compression in human subjects were demonstrated in a 2014 study [28]. The authors reviewed the pathology of resected sections of the lateral femoral cutaneous nerve of the thigh in seven subjects with meralgia paresthetica who underwent neurectomy for chronic, disabling pain. Histology of the resected nerves showed perineural thickening, demyelination, axonal degeneration, and varying degrees of endoneurial and epinueral inflammation. Collections of inflammatory cells of mild-moderate size were observed in five of the seven subjects, and the degree of inflammation correlated with the degree of axonal injury. Review of the same nerve from autopsy controls did not reveal perineural thickening, axonal loss, or inflammation.

Detailed cadaver studies have shown several candidate areas for compression of the ONs along their paths through the posterior cervical muscles and fascia. The GON may be compressed at one or more of many locations: between the semispinalis and the inferior oblique muscle; at the entry to the semispinalis capitis muscle; at the exit from the semispinalis capitis muscle; at the entrance to the trapezius muscle; the exit from the trapezius; at the fascial insertion of the trapezius at the occipital ridge; and by the occipital artery at the distal location of the nerve above the skull base [29]. The lesser occipital nerve (LON) may be compressed by the fascial attachment of the sternocleidomastoid muscle, by branches of the occipital artery, and by fascial bands [30]. At this time, there are not adequate imaging techniques available to image the full scope of the compression sites of the lesser and greater ONs; while segments of the GON may be visualized with ultrasound [31], as well as the morphologic changes caused by compression within the semispinalis capitis muscle [32], it is not possible to visualize the nerve in its entirety to allow determination of all the sites of compression.

We recently reported in Cephalalgia the surgical outcomes of seven patients greater than 3 years following occipital nerve decompression [33]. All patients experienced UHNP with varying presentations; four met criteria for CM, one for CTTH, one for NDPH, and one patient with isolated, posttraumatic occipital pain. All subjects underwent comprehensive decompression of the bilateral LONs and GONs under general anesthesia. Three of the four patients with CM experienced a partial or complete elimination of pain, and each of the subjects with NDPH and posttraumatic occipital pain experienced elimination of pain. Of particular note, while the occipital component of pain was more reliably eliminated after nerve decompression surgery, a reduction in frontal headaches was also experienced in patients with chronic migraine and NDPH [33]. There were no adverse effects of the surgery. Compression resulting in morphological changes of the occipital nerves were observed in all patients, as was brisk perineural inflammation.

### Occipital nerve compression as a cause of UHNP

We hypothesize that anatomic entrapment of the GONs and LONs occurs as the nerves pierce the muscular structures of the posterior neck and the fascial attachments at the occipital ridge. While anatomic positioning of the nerve within the muscles appears to be embryologic, subsequent inflammation develops over time due to axonal compression as well as ischemia of the peripherally placed nerve vasculature, and perhaps intensified by other factors such as trauma in the form of either a direct blow or intense pressure to the occiput, or a stretch injury of the nerves. We further speculate that the combination of progressive inflammation with additional direct compression of the nerve by the accumulating inflammatory tissue and spasm in the surrounding muscle leads to unremitting pain in the suboccipital area of the neck and the occiput, which may also radiate to more distal distributions of the occipital nerves at the vertex or temples. Co-morbid symptoms including occipital allodynia and pain and painful spasm in cervical and trapezius muscles may also develop. Physical activities that engage the trapezius and other cervical muscles, including actions such as cervical flexion or head turn, may aggravate pain. We speculate that pain signals originating extracranially may spread intracranially via branches of trigeminal nerves that have traversed the calvarium and now reside in the inflamed periosteum [34] as well as via upper cervical nerve fibers passing intracranially [14]. This may in turn lead to activation of intracranial meningeal nociceptors that may precipitate frontal radiation of pain, in the temples in the setting of lesser occipital nerve compression, and in the vertex, forehead and retro-orbital location for greater occipital nerve compression; such nociceptive activation may also be responsible for the co-morbid migraine-like symptoms of photophobia and nausea that may occur.

We speculate that while triptan agents may be effective for the acute exacerbations of frontal, trigeminally-mediated pain, the unremitting pain in the neck and occiput often does not respond to such acute interventions; nor does the UHNP generally respond well to centrally directed, membrane stabilizing agents such as anti-convulsants. Peripherally directed treatments such as steroid injections to the occiput in the form of trigger point injections or occipital nerve blocks, as well as botulinum toxin and monoclonal antibodies directed at CGRP, may provide temporary benefit for the UHNP caused by occipital nerve compression. Surgical decompression of the occipital nerves may provide more enduring relief of pain*.*

It is important to note that none of the tenets proposed in this hypothesis conflict with previously reported, evidence-based research in headache pathophysiology. In fact, the outlined hypothesis *aligns* with many previously reported findings. The gradual development of inflammation and progressively worsening compression of the occipital nerves is consistent with the concept of ‘transformation’ of episodic migraine to chronic migraine and may be the cause of chronic migraine particularly in the approximately 34% of patients with CM for whom reversion of CM to EM does not occur. The higher likelihood of allodynia and very high frequency of headache in such patients is also consistent with the concept of ON compression, with the caveat that the reported allodynia is generally occipital and not in the trigeminal distributions, and therefore may not be reliably detected by the Allodynia Symptom Checklist, which is weighted more heavily toward trigeminal distribution symptoms [35]. It is also of note that the only class of pharmacological agents that was found to have a protective effect in the ‘transformation’ from EM to CM was nonsteroidal anti-inflammatory drugs [36], a finding which may support the presence of an inflammatory process underlying the worsening of headaches. In terms of aligning the phenomenon of ON compression as a cause of headache, the ICHD appendix diagnosis of A11.2.5, Headache attributed to cervical myofascial pain, may come closest to capturing the scope of this condition. The criteria include ‘a source of myofascial pain in the muscles of the neck, including reproducible trigger points’ and ‘tenderness…(with) pressure on the implicated cervical muscles.’ We posit that the muscle is not the ultimate generating source of the pain, but rather that the nerve entrapment within the muscle and the resulting inflammation is the cause of pain; the general idea of the pathophysiologic condition being that of a soft tissue abnormality in the neck as the cause of pain is maintained.

It is important and helpful to distinguish the symptoms of UHNP associated with ON compression from the condition of Occipital Neuralgia as defined in the ICHD [3]. Occipital Neuralgia is defined as pain that has two of the following three characteristics: [1] recurring in paroxysmal attacks lasting from seconds to minutes; [2] severe in intensity; [3] shooting, stabbing or sharp in quality. The pain is associated with dysesthesia and/or allodynia, and either tenderness to palpation of the nerve or its branches, or the presence of tender trigger points at the emergence of the GON. The pain must also be temporarily eased by an anesthetic block of the GON.

In our experience and those of other clinicians, Occipital Neuralgia is uncommon. There are some areas of overlap between the clinical presentation of ON compression and Occipital Neuralgia, however, as well as some areas of important difference. Areas of overlap of the two conditions include [1] the presence of allodynia and [2] tenderness to palpation of either the nerve or the soft tissue of the suboccipital space. Areas of difference include [1] the character of pain: most individuals with ON compression report their primary pain symptom to be tightness and pressure, not stabbing, sharp pain (such neuropathic symptoms are occasionally encountered in our [PB] experience, but they are almost always less frequent than the overwhelming pressure sensation); [2] the duration of the symptoms, which as noted repeatedly above, is generally unremitting in ON compression, and not in the paroxysmal attacks required for Occipital Neuralgia; and [3] the intensity of pain, which in ON compression is most often not severe, and may be mild to moderate on a typical day, as opposed to the severe intensity that is required for the diagnosis of Occipital Neuralgia. Finally, we find the response to ON blocks in ON compression (as proven by later response to surgical decompression) to be somewhat variable. While the majority of patients experience a reduction in pain, not all do, and some patients experience a provoked headache that may offset any eventual benefit from the block.

### The spectrum of headache pathophysiology

Occipital nerve compression as the cause of UHNP in no way excludes the presence of clearly central and brainstem processes being instrumental in the pathophysiology of other headache types, such as low-frequency migraine with aura. One may, in fact, conceptualize headache disorders existing along a continuum of pathophysiology, reflecting the anatomic continuum of nerves from intra- to extracranial. Strongly central processes such as low-frequency episodic migraine with aura lie at one end, the “central end,” of the spectrum, while unremitting, solely sub-occipital and occipital pain lies at the other, “peripheral end,” of the spectrum. Most patients will reside at a point between the ends of the spectrum, and some patients may have two completely different headache types, and thus reside at two places on the spectrum. The clinical characteristics of the separate headaches will differ, as will effective treatments for each headache type. The concept of a spectrum for the pathophysiology of headache disorders is, in fact, illustrated by the range of responses following nerve decompression [33]. Some patients with the purely peripheral factor of nerve compression may have a significant reduction in pain, similar to the reduction of lumbar radicular pain seen after a lumbar microdiscectomy, while those individuals with additional centrally-mediated headaches such as menstrual migraine or migraine with aura will likely to continue to experience these headaches. In the authors’ (PB) experience, the correct identification of patients who have predominant peripheral factors such as occipital nerve compression as the cause of UHNP is the most important step in determining if occipital nerve decompression is an appropriate intervention. It is important to note, however, that elimination of the posterior peripheral trigger for UNHP *may* additionally reduce frontal headaches that are consistent with migraine [33], again, likely reflecting the anatomic spectrum of the neural networks that span both the extracranial and intracranial spaces, and the elimination of activation of extracranial trigeminal nociceptors by the removal of the inflammation-causing compression. It is also of note that indirect results of nerve decompression may result in additional improvement that extends beyond the elimination of UNHP, such as improved sleep from the reduction in occipital allodynia.

## Conclusion

Chronic headache remains a leading cause of disability globally [37] and in the United States [38]. In the setting of inadequate treatment responses to centrally-acting interventions, the search for the cause of, and effective treatments for, head and neck pain has moved steadily toward the periphery for years. Accumulating anatomic research and the effectiveness and increasing use of peripherally- and even extracranially-directed treatments speaks to the growing acceptance of the role of peripheral structures in the development of headache. The concept of occipital nerve compression as a cause of UHNP may be considered an extension of this previously existing work. Further study is needed to elucidate the prevalence of UHNP and the mechanism and pathophysiology of ON compression, and research into safe and effective interventions for such should be pursued. Further careful study is needed of the mechanism of occipital nerve compression, the technique of occipital nerve decompression surgery, and the role of the surgery in the clinical practice of Headache Medicine.

## Data Availability

Data sharing is not applicable to this article as no datasets were generated or analyzed during the current study.

## Abbreviations

CGRP: calcitonin gene related peptide
CM: chronic migraine
CTTH: chronic tension-type headache
GON: Greater occipital nerve
HC: Hemicrania Continua
ICHD: International Classification of Headache Disorders
LON: Lesser occipital nerve
NDPH: new daily persistent headache
ON: occipital nerve
UHNP: unremitting head and neck pain
